# Perceived quality teaching and student retention intention: The serial mediation of knowledge, skills, competencies, and student loyalty

**DOI:** 10.1371/journal.pone.0361914

**Published:** 2026-11-02

**Authors:** Shahzaf Iqbal, Che Azlan Bin Taib, Nura Abubakar Allumi, Hussein Abu al-Rejal, Gulshan Fatima Alvi, Md Abdul Kafi, Mazhar Abbas

**Affiliations:** 1 School of Technology Management and Logistics, Universiti Utara Malaysia, Sintok, Kedah, Malaysia; 2 Education Department, The University of Lahore, Pakistan; COMSATS University Islamabad - Wah Campus, PAKISTAN

## Abstract

Student retention intention remains a concern for higher education systems worldwide and is relevant to institutional sustainability and Sustainable Development Goal 4 (SDG 4). Drawing on Human Capital Theory (HCT) and Expectancy–Value Theory (EVT), this study examines the direct and indirect associations between perceived quality teaching and student retention intention through students’ self-reported knowledge, skills, competencies and student loyalty. Cross-sectional survey data were collected from 395 final-year students across Pakistani universities using a multistage sampling approach, with institutions selected through stratified random sampling and participants recruited via convenience sampling. The proposed relationships were examined using structural equation modeling with SmartPLS 4. The results indicate that perceived quality teaching is positively associated with student retention intention, both directly and indirectly through students’ knowledge, skills, and competencies and student loyalty. Significant indirect associations were identified through each mediator separately and through the proposed serial mediation pathway. These findings highlight the relevance of considering students’ perceived capability development and institutional attachment together when examining retention intention. HCT provides a rationale for the relationship between perceived teaching quality and students’ knowledge, skills, competencies, while EVT offers an interpretive perspective; however, expectancy of success and subjective task value were not directly measured. The cross-sectional design also precludes conclusions about temporal ordering or causality. Practically, the findings suggest that faculty development, student-centered pedagogies, digitally supported teaching, and curriculum alignment with labor market needs merit consideration in institutional quality assurance. The findings provide context-specific evidence from Pakistani higher education (HE) and are relevant to SDG 4 through their focus on perceived teaching quality and students’ intentions to continue their studies. They do not establish effects on actual retention or broader educational outcomes.

## 1. Introduction

Student retention intention has become an increasingly important concern in higher education (HE), as institutions are increasingly assessed in terms of learning outcomes, completion rates, and students’ continued intention to persist in their studies. In this context, quality teaching is widely regarded as a key driver of meaningful learning and student success. These priorities align closely with Sustainable Development Goal 4 (SDG 4), which emphasizes inclusive and high-quality education as a foundation for sustainable development. Beyond serving as a performance indicator, student retention intention reflects students’ willingness to continue their studies and serves as an important antecedent of actual student retention behavior. Perceived quality teaching and student retention intention can therefore be understood as interconnected elements within a broader agenda of sustainable HE [1]. However, retention intention is distinct from actual retention, and examining its relationship with teaching quality requires attention to the processes associated with students’ intentions to persist.

Research on student retention intention has traditionally been informed by models of student persistence and dropout, highlighting the roles of academic integration, social engagement, and structural constraints [2–4]. More recent studies have expanded this perspective by incorporating cognitive, emotional, and institutional dimensions of the learning experience [5,6]. Despite these advances, student retention intention remains a persistent challenge, particularly in developing countries where socioeconomic constraints, resource limitations, and disparities in educational quality may shape students’ progression and completion [7]. While service quality and related outcomes have received considerable attention, the mechanisms through which quality teaching contributes to sustained student participation remain insufficiently specified. Empirical evidence indicates that perceived teaching quality is associated with students’ intention to remain enrolled [8]. Related work also associates supportive learning environments and effective teaching practices with student engagement and intrinsic motivation, both of which are relevant to persistence in HE [9]. Nevertheless, these findings leave room to examine how students’ perceptions of their learning-related capabilities and their attachment to an institution jointly relate to retention intention. In this study, students’ self-reported knowledge, skills, and competencies (KSCs) are conceptualized as a unified construct reflecting their perceived development across these related domains. This issue is particularly relevant in developing HE systems, where differences in pedagogical practices and learning environments may influence these relationships [10].

Two interrelated gaps motivate this study. First, further evidence is needed on the indirect relationship between perceived quality teaching and retention intention through students’ self-reported KSCs. Second, cognitive and affective dimensions of students’ educational experiences are often examined separately, providing a limited understanding of how perceived capability development and institutional attachment may operate together. Examining KSCs and student loyalty as separate mediators allows their respective indirect associations with retention intention to be assessed. Examining them serially also tests whether the proposed pathway from KSCs to student loyalty is consistent with the data. The contribution is therefore not simply the inclusion of two mediators, but the examination of their distinct and combined roles within one model.

To address these gaps, this study draws on Human Capital Theory (HCT) and Expectancy-Value Theory (EVT). HCT conceptualizes education as an investment that enhances individuals’ productive capabilities through the development of knowledge and skills [11,12], providing a rationale for examining the relationship between perceived quality teaching and students’ self-reported KSCs. EVT emphasizes the importance of expectations of success and the value attached to activities in motivation and persistence [13]. Because expectancy of success and subjective task value are not directly measured, EVT serves as an interpretive perspective rather than a theory tested in its entirety. Student loyalty is not treated as equivalent to either EVT construct; instead, it represents students’ institution-focused attachment. The proposed sequence—perceived quality teaching → KSCs → student loyalty → retention intention—distinguishes perceived learning-related benefits from institutional attachment. Students who perceive that their education supports the development of useful capabilities may evaluate their educational experience more favorably, which may coincide with stronger loyalty and retention intention. This ordering provides a theoretical rationale for the proposed serial mediation, but it is not an established temporal or causal sequence. Parallel and reciprocal relationships remain plausible alternatives.

The relevance of this investigation is particularly evident in Pakistan’s HE sector. Despite considerable expansion—from 58 higher education institutions (HEIs) in 2000–262 by 2025 [14]—concerns regarding educational quality and student outcomes persist. Evidence points to uneven teaching practices, limited resources, and misalignment between curricula and contemporary skill requirements [15,16], as well as disparities between public and private institutions [17]. At the same time, sustainability remains only partially embedded in core academic functions, including curriculum design and faculty development [18,19]. These issues provide context for examining the relationships among perceived quality teaching, KSCs, student loyalty, and retention intention in Pakistan; they do not establish that these conditions apply uniformly across institutions or that the proposed relationships are unique to this setting.

Against this backdrop, the present study examines the relationship between perceived quality teaching and student retention intention through the direct and indirect associations involving students’ self-reported KSCs and student loyalty. It makes three contributions. First, it examines the separate indirect associations through KSCs and student loyalty alongside their proposed serial mediation, providing a more differentiated account of how perceived capability development and institutional attachment relate to retention intention. Second, it brings HCT and EVT into a common analytical framework while distinguishing the relationships examined from EVT mechanisms that were not directly measured. Third, it provides empirical evidence from Pakistani HE, extending investigation of these relationships to this context. The findings are relevant to SDG 4 because they concern perceived teaching quality and students’ intentions to continue their education; they do not directly demonstrate improvements in educational equity, completion rates, or actual retention. Accordingly, this study addresses the following research question (RQ):

**RQ:** How are perceived quality teaching and student retention intention related, both directly and indirectly through students’ self-reported knowledge, skills, and competencies and student loyalty, in Pakistani universities?

The remainder of the paper is organized as follows. The next section reviews the literature and develops the study’s hypotheses, followed by the methodology and results. The final section presents the discussion, including theoretical contributions, empirical evidence, and practical and policy implications, followed by the conclusion, limitations, and directions for future research.

## 2. Theoretical foundations

This study is grounded in Human Capital Theory (HCT) and Expectancy-Value Theory (EVT), which offer complementary perspectives for examining the relationships among perceived quality teaching, students’ knowledge, skills, and competencies, student loyalty, and student retention intention. HCT provides the primary rationale for the proposed capability-related pathway, while EVT offers a perspective for interpreting students’ evaluations of their educational experiences. Because expectancy of success and subjective task value are not directly measured, EVT serves as an interpretive lens rather than a theory tested in its entirety.

HCT conceptualizes education as an investment that enhances individuals’ productive capabilities through the development of knowledge, skills, and competencies [11,12]. In this study, students’ knowledge, skills, and competencies are conceptualized as a unified construct reflecting students’ self-perceived development across these related domains. Within HE, perceived quality teaching may be associated with students’ perceptions of their learning and capability development. However, because students’ knowledge, skills, and competencies are self-reported, they represent perceived rather than objectively assessed human capital. HCT helps explain the proposed relationship between perceived quality teaching and students’ knowledge, skills, competencies, but does not fully account for students’ institutional loyalty or retention intention. EVT complements this perspective by proposing that motivation and persistence are related to expectations of success and the value individuals attach to learning experiences [13].

The proposed relationship between students’ knowledge, skills, competencies and student loyalty draws on the distinction between perceived learning-related benefits and students’ attachment to their institution. Students who perceive that their education supports the development of useful capabilities may evaluate their educational experience more favorably, which may coincide with stronger institutional loyalty. Previous research provides a basis for considering this relationship [20,21]. Nevertheless, student loyalty is not equivalent to expectancy of success or subjective task value; consequently, the present study does not establish that these specific EVT mechanisms explain the relationship.

The proposed sequence—perceived quality teaching → students’ knowledge, skills, and competencies → student loyalty → retention intention—places perceived capability development before institution-focused attachment. This ordering reflects the theoretical proposition that students’ perceptions of what they gain from learning may inform their broader evaluation of the educational experience and their institutional commitment. Student loyalty may, in turn, be associated with their intention to continue studying. However, this sequence is not necessarily preferable in all circumstances to parallel or alternative pathways. Loyalty may develop directly from teaching experiences or institutional factors, and existing loyalty may also shape students’ perceptions of teaching and learning. The serial mediation model therefore tests one theoretically informed arrangement of these relationships rather than establishing a temporal or causal sequence.

Taken together, HCT and EVT provide complementary, but not fully tested, explanations for examining the direct and indirect associations among the study variables. The study’s contribution lies in investigating the separate and serial mediating roles of students’ knowledge, skills, and competencies and student loyalty in Pakistani HE, rather than claiming to extend or refine EVT. Its relevance to SDG 4 concerns the relationships among perceived teaching quality, students’ perceived development (knowledge, skills, competencies), and retention intention. The conceptual model is presented in Fig 1.

**Fig 1:**
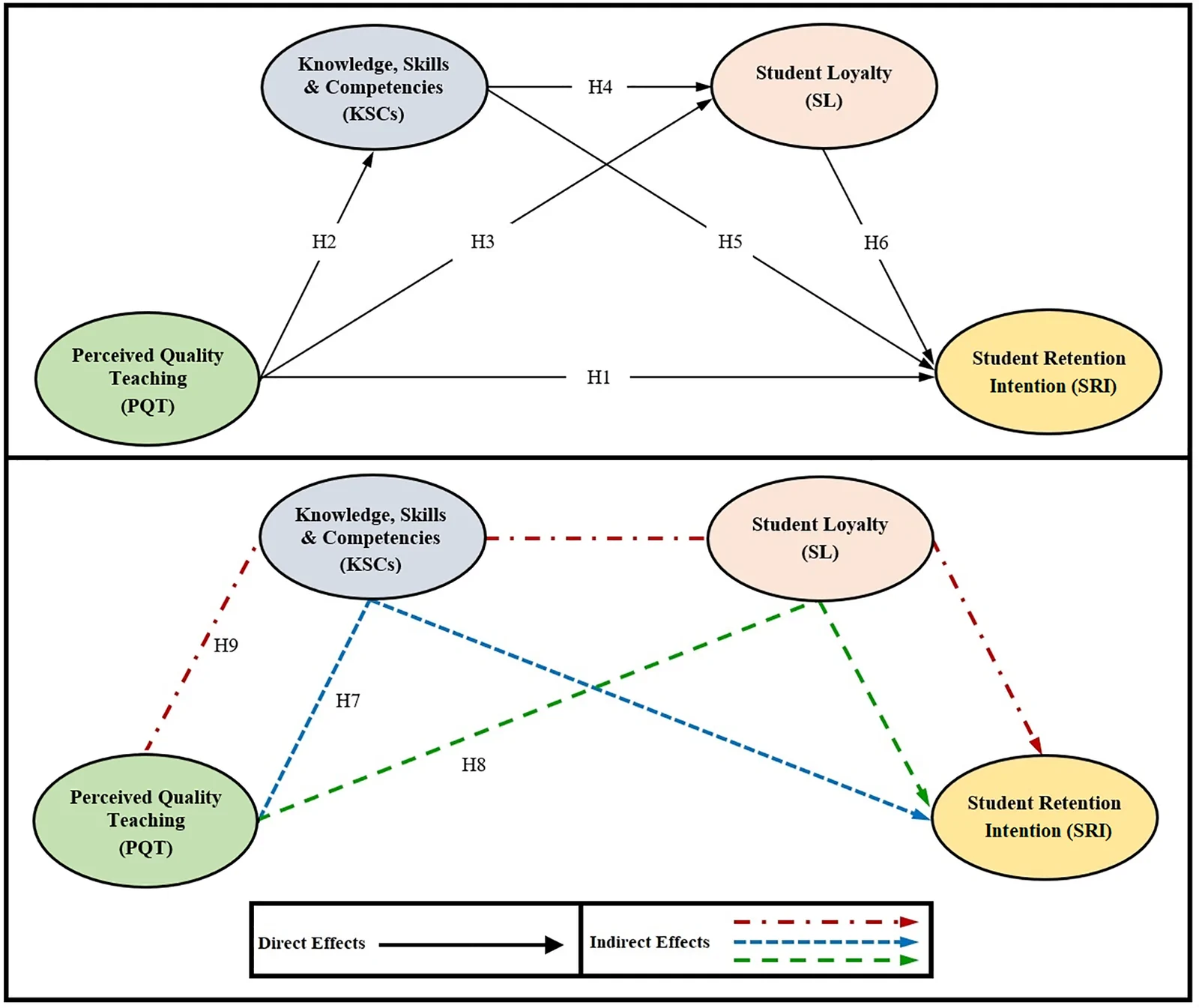
Conceptual framework.

## 3. Hypotheses development

### 3.1 Perceived quality teaching and student retention intention

Perceived quality teaching is widely recognized as a central determinant of student retention intention in HE, primarily through its influence on students’ academic engagement and learning experiences. Research consistently shows that instructional clarity, learner support, relevance, and feedback enhance academic performance and reduce attrition by strengthening both cognitive development and emotional engagement [21,22]. However, literature also suggests that perceived quality teaching is often embedded within broader institutional reform agendas, which can obscure its distinct explanatory role in student retention intention [23]. Across studies, student engagement and perceived faculty support emerge as key mechanisms linking perceived quality teaching to continued enrollment, reflecting both cognitive and affective pathways of influence [9,24,25]. In developing contexts such as Pakistan, these dynamics are further shaped by uneven instructional quality, limited faculty preparedness, and resource constraints, which collectively weaken the effectiveness of teaching practices [26]. Although attrition is often attributed to socioeconomic and cultural factors, recent evidence increasingly positions perceived quality teaching as an equally critical explanatory factor [27], particularly in systems where structural limitations persist despite policy reforms [15,28,29]. Building on these insights, the study develops the following hypothesis.

**H1**: Perceived quality teaching has a positive effect on student retention intention.

### 3.2 Perceived quality teaching and its relationships with students’ knowledge, skills and competencies and student loyalty

Perceived quality teaching is central to the development of students’ knowledge, skills, and competencies and student loyalty. High-quality instruction fosters cognitive, interpersonal, and behavioral growth, with pedagogical competencies—such as curriculum design, clarity, and interactive teaching—linked to improved learning outcomes [30]. Yet, the long-term effects of these competencies remain underexplored [31], particularly within Pakistani HEIs, where most evidence originates from secondary education [32,33]. Student loyalty, reflecting students’ emotional attachment and commitment to their institution, is also shaped by perceived quality teaching. While student satisfaction has been widely examined as an outcome of teaching quality, its direct influence on loyalty is less studied [34]. In Pakistan, empirical support exists but is largely confined to private universities, limiting generalizability. Additional factors such as institutional image, perceived value, and trust also affect student loyalty [35], while faculty engagement has been shown to foster both satisfaction and loyalty [36]. Although prior research confirms the role of perceived quality teaching in enhancing student satisfaction, its capacity to build enduring student loyalty remains insufficiently examined [37]. Therefore, this study examines how perceived quality teaching shapes students’ knowledge, skills, and competencies and student loyalty, positioning them as key mechanisms influencing student retention intention. This leads to the following hypotheses.

**H2**: Perceived quality teaching has a positive effect on students’ knowledge, skills, and competencies.

**H3**: Perceived quality teaching has a positive effect on student loyalty.

### 3.3 Students’ knowledge, skills, competencies and student loyalty

The development of students’ self-reported knowledge, skills, and competencies—including communication, disciplinary competence, teamwork, analytical, organizational, and entrepreneurial abilities—is central to fostering both student loyalty and student retention intention in HE. When students perceive their curriculum and institutional activities as enhancing these competencies, they report stronger confidence, perceived value, and emotional attachment to their institution. Prior research shows that perceived competence improves behavioral control and satisfaction, which are important antecedents of student loyalty [38]. Similarly, academic service quality that promotes learning and development significantly influences student loyalty through student satisfaction [39], while faculty–student interactions that enhance competence predict loyalty intentions [40]. However, most existing studies embed knowledge, skills, and competencies within broader constructs such as service quality, rarely isolating their direct effect. This gap highlights the need to examine how improvements in targeted competencies—such as communication, teamwork, and problem-solving—independently strengthen student loyalty. Given that student loyalty fosters emotional attachment that supports students’ intention to persist, examining this pathway is particularly important in developing countries where student retention intention remains a pressing concern. Accordingly, the following hypothesis is proposed.

**H4**: Students’ self-reported knowledge, skills, and competencies have a positive effect on student loyalty.

### 3.4 Students’ knowledge, skills, competencies and student retention intention

Students’ self-reported knowledge, skills, and competencies are also positively associated with student retention intention, as the development of relevant competencies strengthens students’ confidence, motivation, and commitment to academic progression. Conversely, perceived deficits in communication, problem-solving, teamwork, or organizational skills may weaken students’ intention to persist and reduce their intention to remain enrolled. Research shows that innovative teaching approaches, such as problem-based learning, enhance critical thinking and reduce attrition [23]. In Pakistan, student retention intention is influenced by academic readiness, teaching quality, and institutional support structures [41, 42], while sociocultural factors also contribute to early withdrawal [27]. Yet, few studies isolate the role of knowledge, skills, and competencies in predicting student retention intention, often treating them as background variables rather than direct predictors. Addressing this gap, the present study proposes the following hypothesis:

**H5**: Students’ self-reported knowledge, skills, and competencies have a positive effect on student retention intention.

### 3.5 Student loyalty and student retention intention

Student loyalty is a crucial determinant of student retention intention, reflecting students’ emotional attachment and long-term commitment to their institution. Prior research shows that loyalty, strengthened by satisfaction, perceived value, and engagement, lowers dropout risk [43]. Institutional image, trust, and reputation also enhance both student loyalty and student retention intention [35,37,44]. While international studies emphasize institutional drivers of loyalty, research in Pakistan has primarily focused on academic quality and support, with limited attention to individual attributes such as self-efficacy and socio-cultural influences. Moreover, the growing role of digital learning in shaping student loyalty and student retention intention remains underexplored [45]. Consequently, the following hypothesis is proposed:

**H6**: Student loyalty has a positive effect on student retention intention.

### 3.6 The serial mediating roles of students’ knowledge, skills, and competencies and student loyalty

Perceived quality teaching is increasingly recognized as an important correlate of student retention intention in HE, with students’ self-reported knowledge, skills, and competencies and student loyalty representing distinct potential pathways. Effective instruction may support transferable capabilities, including critical thinking, communication, and adaptability, which are relevant to academic and professional preparation [46,47]. Pedagogical innovations have also been associated with competency development [48]. However, these findings do not establish the extent to which students’ knowledge, skills, and competencies mediate the relationship between perceived quality teaching and retention intention. Although related relationships have been examined [29,49], this indirect pathway remains insufficiently investigated in the present context.

Similarly, student loyalty, although widely studied as an outcome of academic experience, has received less attention as a mediator between perceived quality teaching and retention intention. Favorable teaching experiences and perceived educational value may be associated with students’ emotional attachment and commitment to their institution [50,51]. Student loyalty has also been linked to trust, perceived value, and institutional image [52], but its mediating role remains underexplored in contexts such as Pakistan [53]. Examining students’ knowledge, skills, and competencies and loyalty separately distinguishes students’ perceptions of capability development from their institutional attachment, allowing their respective indirect relationships with retention intention to be assessed.

These two pathways also provide a rationale for examining serial mediation. Students who perceive that teaching supports their development of useful knowledge, skills, and competencies may evaluate the value of their educational experience more favorably, which may be associated with stronger institutional loyalty. This proposed sequence distinguishes capability-related considerations from affective attachment and provides a theoretical basis for examining their joint indirect relationship with retention intention. However, loyalty may also develop directly from teaching experiences or institutional reputation, while existing loyalty may shape students’ evaluations of teaching and their perceived capability development. Such reciprocal relationships and alternative pathways cannot be ruled out. The proposed ordering is therefore a theoretically informed hypothesis, not an established temporal or causal sequence. To address these gaps, the following mediation hypotheses are proposed:

**H7:** Students’ knowledge, skills, and competencies mediates the relationship between perceived quality teaching and student retention intention.

**H8:** Student loyalty mediates the relationship between perceived quality teaching and student retention intention.

**H9:** Students’ knowledge, skills, and competencies and student loyalty sequentially mediate the relationship between perceived quality teaching and student retention intention.

## 4. Methodology

### 4.1 Sample and data collection

This study employed a cross-sectional survey design to collect data from final-year undergraduate and postgraduate students across Pakistani universities. The design is appropriate for capturing students’ perceptions at a single point in time and for examining associations among the study variables [54,55]. However, the cross-sectional design does not establish temporal ordering or causal relationships. A multistage sampling strategy was adopted, combining non-proportionate stratified random sampling with convenience sampling, consistent with prior research [56–58]. Universities were first stratified by region, with one public and one private institution randomly selected from each province, along with one institution (public or private) from each administrative unit. Participants were then recruited through convenience sampling to facilitate access under logistical constraints. This approach is suitable for geographically dispersed populations where a complete sampling frame is not readily available, thereby facilitating participant access under practical constraints [59–61]. Nevertheless, because convenience sampling was used at the student level, the final sample was not a probability sample of Pakistani university students, and the findings should not be interpreted as fully representative of the wider student population.

Using this approach, 550 questionnaires were distributed across 11 universities between 1 February 2025 and 30 April 2025. A total of 415 responses were received; after excluding 20 cases due to straight-lining, 395 valid questionnaires were retained for analysis, yielding an effective response rate of 71.8%. The distribution of respondents across the participating universities and their demographic characteristics is reported in Table 2. Sample size adequacy was assessed using *a priori* power analysis in G*Power, assuming a medium effect size (f^2^ = 0.15), (alpha = 0.05), and statistical power of 0.95. The analysis indicated a minimum required sample of 119 respondents; thus, the final sample of 395 exceeded this threshold [62,63]. Although the sample exceeded the estimated minimum, this calculation does not account for potential dependence among students from the same university. Students within an institution may share teaching practices, institutional policies, infrastructure, and support systems, which may affect the precision of the estimated relationships.

Ethical approval was obtained from the University of Lahore, Pakistan (Institutional Research Ethics Board; IREB Letter No. UOL/IREB/25/8.1/0001). Data collection was conducted in accordance with the approved research protocol. Electronic informed consent was obtained from all participants before they accessed the questionnaire. Participants were informed about the study’s purpose, the voluntary nature of participation, their right to withdraw at any time without penalty, and the confidentiality and anonymity of their responses.

### 4.2 Measures

This study employed a structured survey instrument comprising 30 items divided into two main sections. Data were collected using Google Forms and distributed through faculty networks. Responses were recorded on a five-point Likert scale ranging from 1 (“Strongly Disagree”) to 5 (“Strongly Agree”), with 3 indicating a neutral position. The first section gathered demographic information through six items, while the second section included 24 items adapted from established research. Specifically, five items assessed perceived quality teaching [64–66], while 11 items measured students’ knowledge, skills, and competencies [67], which were specified as a single latent construct intended to represent students’ perceived development across these three related domains. As the measure relied on self-reports, it captured students’ perceptions of their capabilities rather than objectively assessed learning outcomes. Student loyalty was evaluated using three items [68], and student retention intention was measured with five items [69] to capture students’ stated intentions to continue their studies rather than actual retention behavior. To assess content validity and contextual relevance, the instrument underwent expert review involving three faculty members and two quality assurance professionals from Pakistani universities. Based on their input, several items were revised for improved clarity and alignment with the study context.

## 5. Data analysis and results

Data analysis was conducted in two phases. Preliminary analyses were performed using SPSS (Version 25), where the Kolmogorov–Smirnov test indicated that the data were not normally distributed. Although this result was considered when selecting the analytical approach, non-normality alone does not determine the choice of SEM method. Given the cross-sectional, single-source survey design, common method bias (CMB) and multicollinearity were subsequently assessed. All variance inflation factor (VIF) values (see Table 1) were below the recommended threshold of 3.3, indicating that the full-collinearity assessment did not identify serious concerns regarding CMB [70]. However, these results do not establish the absence of CMB. In addition, all VIF values were below 3.0, suggesting that problematic multicollinearity was not indicated under the criterion applied [71]. Following these preliminary assessments, partial least squares structural equation modeling (PLS-SEM) was performed using SmartPLS 4. PLS-SEM was selected because the study examines relationships among latent constructs and assesses direct and indirect associations within the proposed serial mediation model. Unlike conventional maximum-likelihood estimation in covariance-based SEM, PLS-SEM does not require the same multivariate normality assumptions, making it suitable for analyzing models involving non-normally distributed data. which is well suited to non-normal data and complex models. Bootstrapping was employed to estimate the significance of the model parameters and indirect effects, providing a means of assessing the statistical significance of the hypothesized relationships and indirect effects without relying on conventional normal-theory tests [72].

**Table 1:** Full-Collinearity test.

Criterion Variable	Predictor Variables	Tolerance	VIF
PQT	KSCs	0.470	2.127
	SL	0.483	2.072
	SRI	0.408	2.453
KSCs	SL	0.512	1.954
	SRI	0.411	2.432
	PQT	0.558	1.791
SL	SRI	0.458	2.182
	PQT	0.456	2.194
	KSCs	0.407	2.457
SRI	PQT	0.493	2.028
	KSCs	0.419	2.388
	SL	0.587	1.703

**Note:** PQT = perceived quality teaching; KSCs = knowledge, skills and competencies; SL = student loyalty; SRI = student retention intention.

**- Source:** Authors’ own findings.

### 5.1 Respondent profile

A total of 550 questionnaires were distributed to final-year undergraduate and postgraduate students across 11 universities in Pakistan (see Table 2). Of these, 415 were returned, and after removing 20 responses exhibiting straight-lining behavior, 395 valid questionnaires were retained for analysis, yielding an effective response rate of 71.8%. Eligibility was verified through screening questions, and informed consent was obtained from all participants prior to data collection. The sample included 53.7% of respondents from public and 46.3% from private institutions, with the highest representation from Punjab (19.7%), Sindh (19.2%), and Balochistan (18.7%). Gender distribution was nearly balanced (51.4% male, 48.6% female), and most participants were aged 26–30 (35.4%), followed by those 25 or younger (31.6%). In terms of qualifications, 46.3% were BS, 42.5% MS/MPhil, and 11.1% PhD students. Management Sciences (32.2%), Social Sciences (31.6%), and Computer Sciences (27.1%) were the most represented disciplines. These figures describe the composition of the participating sample; given the use of convenience sampling at the student level, they should not be interpreted as evidence that the sample is fully representative of Pakistani university students.

**Table 2:** Respondents profile.

Items	(N)	%
University ID/Code		
University-A	32	8.10
University-B	34	8.61
University-C	40	10.13
University-D	30	7.59
University-E	34	8.61
University-F	36	9.11
University-G	35	8.86
University-H	41	10.38
University-I	37	9.37
University-J	39	9.87
University-K	37	9.37
Sector		
Public Sector	212	53.7
Private Sector	183	46.3
Province/ Admin. Areas		
Azad Jammu Kashmir	32	8.1
Balochistan	74	18.7
Gilgit Baltistan	30	7.6
Islamabad	34	8.6
Khyber Pakhtunkhwa	71	18.0
Punjab	78	19.7
Sindh	76	19.2
Gender		
Male	203	51.4
Female	192	48.6
Age		
25 Years or Less	125	31.6
26 - 30 Years	140	35.4
31 - 35	79	20.0
36 - 40	28	7.1
41 and More	23	5.8
Qualification		
BS	183	46.3
MS/M.Phil	168	42.5
PhD.	44	11.1
Department		
Social Sciences	125	31.6
Management Sciences	127	32.2
Computer Sciences	107	27.1
Natural Sciences	36	9.1

**Source:** Authors’ own findings.

### 5.2 Descriptive statistics

Descriptive statistics (see Table 3) indicated relatively high mean scores across all study variables, with mean scores ranging from 3.717 for students’ knowledge, skills, and competencies to 3.843 for student retention intention. Perceived quality teaching had a mean of 3.812, while student loyalty had a mean of 3.736. Standard deviations ranged from 0.539 to 0.688, indicating some variation in respondents’ ratings across the variables. Student retention intention had the highest mean score, whereas students’ knowledge, skills, and competencies had the lowest.

**Table 3:** Descriptive Statistics.

	N	Minimum	Maximum	Mean	Std. Deviation (SD)
**PQT**	395	1.60	5.00	3.812	0.688
**KSCs**	395	2.00	4.82	3.717	0.539
**SL**	395	2.00	5.00	3.736	0.632
**SRI**	395	2.20	5.00	3.843	0.664

**Notes:** PQT = perceived quality teaching; KSCs = knowledge, skills and competencies; SL = student loyalty; SRI = student retention intention.

**Source:** Authors’ own findings.

### 5.3 Measurement model

In PLS-SEM, the first step involved evaluating the measurement model for reliability and validity (see Fig 2). As shown in Table 4, all constructs demonstrate acceptable internal consistency, with Cronbach’s alpha (CA) values ranging from 0.703 (for student loyalty) to 0.881 (for students’ knowledge, skills, and competencies), and composite reliability (CR) values between 0.835 and 0.904, exceeding the recommended threshold of 0.70. Convergent validity was confirmed, with average variance extracted (AVE) values above 0.50 for all constructs: perceived quality teaching = 0.651, students’ knowledge, skills, and competencies = 0.511, student loyalty = 0.627, and student retention intention = 0.611. Two items measuring students’ knowledge, skills, and competencies (KSC2 and KSC10) were removed due to low factor loadings. Item KSC11 (0.684) was retained based on theoretical relevance and acceptable convergent validity, as indicated by the overall AVE [73].

**Table 4:** Outer loadings, reliability and convergent validity.

Constructs	Item	Loading	CA	CR	AVE
Perceived quality teaching (PQT)	PQT1	0.813	0.866	0.903	0.651
	PQT2	0.803			
	PQT3	0.787			
	PQT4	0.780			
	PQT5	0.849			
Knowledge, Skills and Competencies (KSCs)	KSC1	0.713	0.881	0.904	0.511
	KSC3	0.724			
	KSC4	0.725			
	KSC5	0.704			
	KSC6	0.750			
	KSC7	0.739			
	KSC8	0.716			
	KSC9	0.677			
	KSC11	0.684			
Student Loyalty (SL)	SL1	0.807	0.703	0.835	0.627
	SL2	0.776			
	SL3	0.793			
Student Retention Intention (SRI)	SRI1	0.787	0.841	0.887	0.611
	SRI2	0.765			
	SRI3	0.807			
	SRI4	0.770			
	SRI5	0.778			

**Note:** AVE, average variance extracted; CR, composite reliability. **Source:** Authors’ own findings.

**Fig 2:**
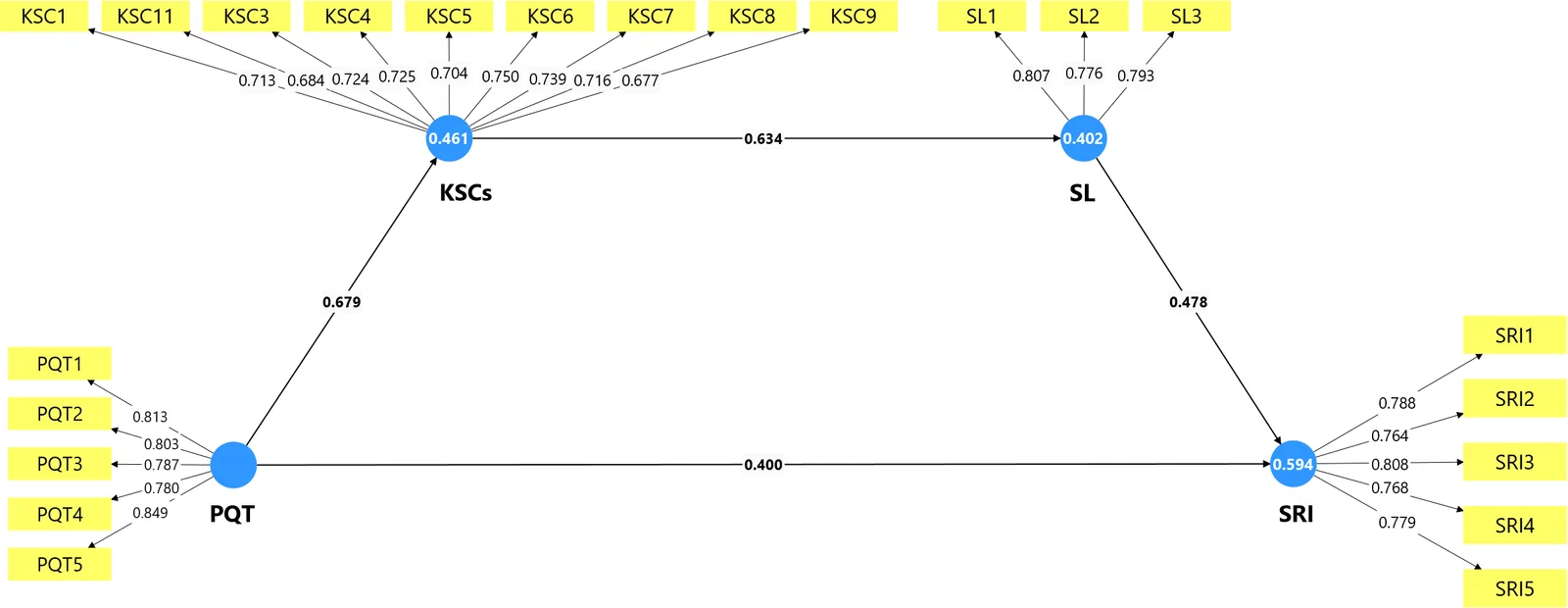
Measurement model.

Discriminant validity was assessed using the Fornell–Larcker criterion and the heterotrait–monotrait ratio (HTMT) (see Table 5). The square roots of the average variance extracted (AVE) exceeded the corresponding inter-construct correlations. Most HTMT values were below the conservative threshold of 0.85. However, the value between student loyalty and student retention intention (0.896) exceeded this threshold, although it remained below the more liberal threshold of 0.90, as discussed in established guidelines [74–76]. The reported 5th and 95th percentile bounds (0.848–0.941) nevertheless extended above 0.90, warranting caution when interpreting the empirical distinction between these constructs. Taken together, the results provide evidence supporting discriminant validity under the criteria considered, although some uncertainty remains regarding the distinctiveness of student loyalty and student retention intention.

**Table 5:** Discriminant Validity.

Fornell–Larcker criterion	PQT	KSCs	SL	SRI
**PQT**	**0.807**			
**KSCs**	0.679	**0.715**		
**SL**	0.537	0.634	**0.792**	
**SRI**	0.657	0.681	0.693	**0.782**
**HTMT criterion**	**PQT**	**KSCs**	**SL**	**SRI**
**PQT**				
**KSCs**	0.751			
**SL**	0.684	0.794		
**SRI**	0.763	0.780	0.896	

**Notes:** CA = Cronbach’s alpha; CR = composite reliability; AVE = average variance extracted; PQT, perceived quality teaching; KSCs, knowledge, skills and competencies; SL, student loyalty; SRI, student retention intention.

**Source:** Authors’ own findings.

### 5.4 Structural model

In the second stage, the structural model (Fig 3) was assessed to evaluate nine hypotheses—six direct (H1–H6) and three indirect (H7–H9)—using bootstrapping with 10,000 subsamples and one-tailed bias-corrected confidence intervals [77]. All reported p-values were less than 0.001 (Table 6). Perceived quality teaching was positively associated with student retention intention (β = 0.281, t = 7.188, CI [0.215, 0.344]), students’ knowledge, skills, and competencies (β = 0.676, t = 23.906, CI [0.626, 0.719]), and student loyalty (β = 0.203, t = 4.205, CI [0.125, 0.282]), supporting H1–H3. Students’ self-reported knowledge, skills, and competencies were positively associated with student loyalty (β = 0.496, t = 10.934, CI [0.419, 0.569]) and student retention intention (β = 0.246, t = 4.620, CI [0.158, 0.334]), supporting H4 and H5. Student loyalty was also positively associated with student retention intention (β = 0.386, t = 9.544, CI [0.318, 0.451]), supporting H6.

**Table 6:** Hypotheses Testing Results.

Hypothesis	Path	β	*t*-value	*p*-value	95% CI [LL, UL]	Result
**Direct Effects**						
H1	PQT → SRI	0.281	7.188	0.000	[0.215, 0.344]	Supported
H2	PQT → KSCs	0.676	23.906	0.000	[0.626, 0.719]	Supported
H3	PQT → SL	0.203	4.205	0.000	[0.125, 0.282]	Supported
H4	KSCs → SL	0.496	10.934	0.000	[0.419, 0.569]	Supported
H5	KSCs → SRI	0.246	4.620	0.000	[0.158, 0.334]	Supported
H6	SL → SRI	0.386	9.544	0.000	[0.318, 0.451]	Supported
**Specific Indirect Effects**						
H7	PQT → KSCs → SRI	0.166	4.594	0.000	[0.108, 0.227]	Supported
H8	PQT → SL → SRI	0.078	3.975	0.000	[0.049, 0.114]	Supported
H9	PQT → KSCs → SL → SRI	0.129	6.612	0.000	[0.100, 0.164]	Supported
**Total Indirect Effects**						
–	PQT → SL	0.335	9.664	0.000	[0.279, 0.393]	Significant
–	PQT → SRI	0.374	11.333	0.000	[0.320, 0.429]	Significant
–	KSCs → SRI	0.192	7.150	0.000	[0.150, 0.239]	Significant
**Total Effects**						
–	PQT → SL	0.538	15.134	0.000	[0.475, 0.593]	Significant
–	PQT → SRI	0.655	23.318	0.000	[0.604, 0.697]	Significant
–	KSCs → SRI	0.438	9.710	0.000	[0.361, 0.510]	Significant

**Note:** CI = confidence interval; LL = lower limit; UL = upper limit; PQT = perceived quality teaching; KSCs = knowledge, skills, and competencies; SL = student loyalty; SRI = student retention intention.

**Source:** Authors’ own findings.

**Fig 3:**
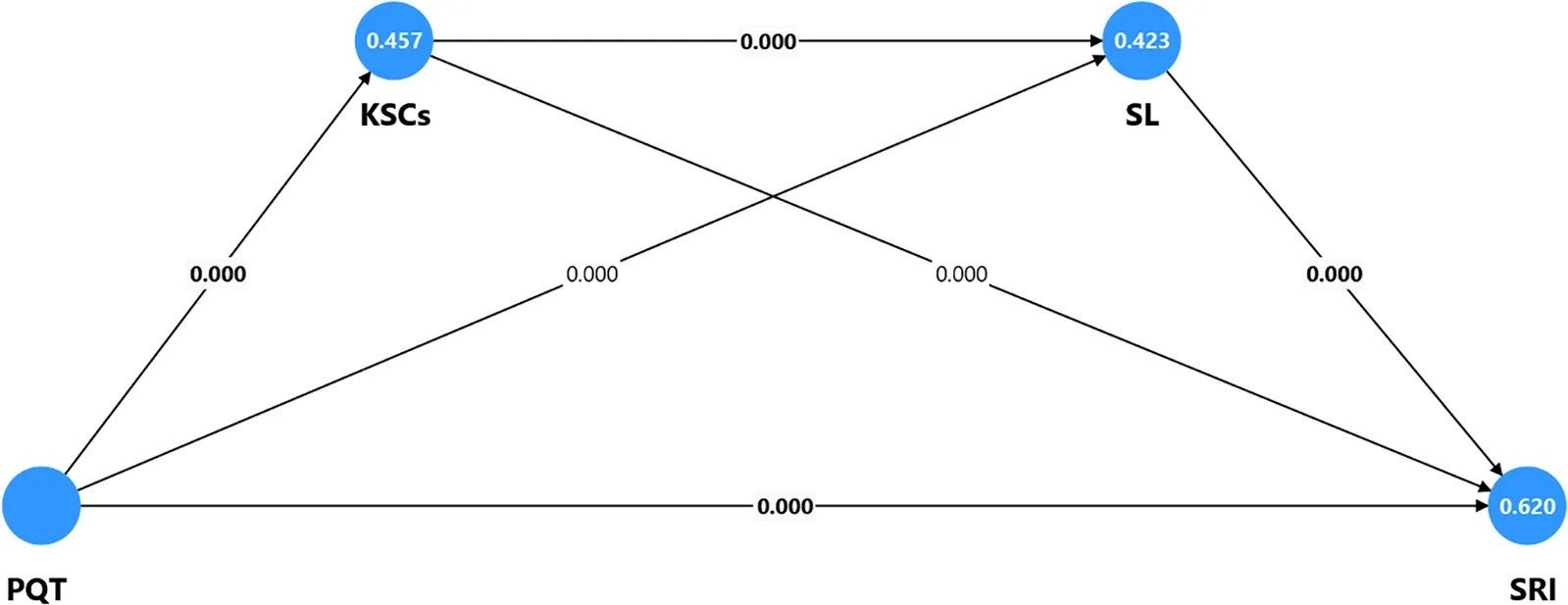
Structural model.

All specific indirect effects were significant: perceived quality teaching → students’ knowledge, skills, and competencies → student retention intention (β = 0.166, t = 4.594, CI [0.108, 0.227]); perceived quality teaching → student loyalty → student retention intention (β = 0.078, t = 3.975, CI [0.049, 0.114]); and perceived quality teaching → students’ knowledge, skills, and competencies → student loyalty → student retention intention (β = 0.129, t = 6.612, CI [0.100, 0.164]), supporting H7–H9. These results indicate statistically significant indirect associations consistent with the proposed mediation pathways; however, the cross-sectional design does not establish their temporal ordering or causal sequence.

The total indirect effect of perceived quality teaching on student retention intention was significant (β = 0.374, t = 11.333, CI [0.320, 0.429]), while the total effect remained strong (β = 0.655, t = 23.318, CI [0.604, 0.697]). Because the direct association also remained statistically significant, the results are consistent with partial mediation under the specified model [78,79]. The significant total effects of perceived quality teaching on student loyalty (β = 0.538) and students’ knowledge, skills, and competencies on student retention intention (β = 0.438) further indicate that the relationships may involve multiple indirect pathways. These findings should be interpreted as associations within the proposed model rather than evidence of a strictly sequential causal process.

Moreover, the explanatory and predictive power of the model was assessed. As shown in Table 7, the coefficient of determination (R²) values indicated that perceived quality teaching explained 46.1% of the variance in students’ knowledge, skills, and competencies, 40.2% in student loyalty, and 59.4% in student retention intention, reflecting moderate to substantial explanatory power [73]. Effect size (f²) estimates, based on [80] guidelines, revealed a very large effect of perceived quality teaching on students’ knowledge, skills, and competencies (0.857), a moderate effect on student retention intention (0.281), and a large effect size for the relationship between students’ knowledge, skills, and competencies and student loyalty (0.673). Student loyalty also showed a large effect size in relation to student retention intention (0.400). Q²_predict values confirmed predictive relevance for students’ knowledge, skills, and competencies (0.454), student loyalty (0.286), and student retention intention (0.428), indicating predictive relevance under the criterion applied [72]. PLSpredict analysis [81] further showed mixed predictive performance relative to the linear-model (LM) benchmark. PLS-SEM RMSE values were lower for 3 of 17 indicators, higher for 13, and equal for 1; MAE values were lower for 10 indicators and higher for 7. Thus, although the model demonstrated predictive relevance, it did not consistently outperform the LM benchmark, particularly in terms of RMSE. Overall, the results provide evidence of explanatory capability, while the model’s comparative predictive performance should be interpreted cautiously.

**Table 7:** Model’s explanatory and predictive performance.

Construct	R²	f² (PQT →)	f² (KSCs →)	f² (SL →)	Q²_predict	RMSE	MAE
KSCs	0.461	0.857	–	–	0.454	0.743	0.619
SL	0.402	–	0.673	–	0.286	0.850	0.689
SRI	0.594	0.281	–	0.400	0.428	0.761	0.618

**Notes:** R² = coefficient of determination; f² = effect size; Q²_predict = predictive relevance; RMSE = root mean square error; MAE = mean absolute error. PQT = perceived quality teaching; KSCs = knowledge, skills, and competencies; SL = student loyalty; SRI = student retention intention.

**Source:** Authors’ own findings.

## 6. Discussion

This study examined the associations among perceived quality teaching, students’ knowledge, skills, and competencies, student loyalty, and student retention intention among final-year students in Pakistan, drawing on Human Capital Theory (HCT) and Expectancy-Value Theory (EVT). The findings offer insights into student persistence and the quality of HE in relation to Sustainable Development Goal 4 (SDG 4). Of the nine hypotheses, six examined direct associations and three examined indirect associations.

The findings indicate that perceived quality teaching is positively associated with student retention intention (H1), consistent with evidence that instructional clarity, relevance, and engagement are associated with academic integration and lower dropout intentions [23,82]. However, the association between perceived quality teaching and student retention intention (β = .281) was weaker than its association with perceived knowledge, skills, and competencies (β = .676). One possible explanation is that students may recognize the immediate learning benefits of effective teaching more directly than its implications for their longer-term institutional decisions. Retention intentions may also reflect factors beyond teaching, including institutional resources and students’ personal circumstances. These considerations are particularly relevant to resource-constrained Pakistani HEIs, where institutional capacity and resource distribution may shape students’ educational experiences [15,26,29].

Perceived quality teaching was also found to be positively associated with both students’ knowledge, skills, and competencies (H2) and student loyalty (H3). The stronger association with perceived knowledge, skills, and competencies suggests that students’ perceptions of teaching quality were more closely aligned with their perceived learning and competency development than with their institutional loyalty. Clear instruction, relevant learning activities, and opportunities to apply knowledge may help students recognize the development of transferable competencies, including communication, teamwork, and analytical reasoning [46,47]. By comparison, loyalty may depend on a broader set of experiences, such as institutional identification and emotional attachment, beyond classroom teaching alone [34,36]. Thus, the results indicate that perceived teaching quality is associated with both cognitive and affective student outcomes, although the strength of these associations differs.

Students’ self-reported knowledge, skills, and competencies were positively associated with student loyalty (H4) and student retention intention (H5). The association between perceived knowledge, skills, and competencies and student loyalty (β = .496) suggests that students who perceive greater development of their capabilities may also evaluate their institutional experience more favorably. The association between perceived knowledge, skills, and competencies and student retention intention was comparatively smaller (β = .246), indicating that perceived competency development may be relevant to persistence intentions without fully accounting for them. These findings extend Pakistan-focused research on academic preparedness [41,42] by highlighting the relevance of students’ perceived competencies to institutional loyalty and retention intentions. However, because perceived knowledge, skills, and competencies were self-reported, the results concern students’ perceptions of their development rather than objectively assessed competencies [38–40].

Similarly, student loyalty was positively associated with student retention intention (H6). The association between student loyalty and student retention intention (β = .386) indicates that loyalty was a comparatively strong correlate of retention intention. Students who feel attached to and identify with their institution may be more inclined to consider continuing their studies there. This interpretation is consistent with research emphasizing the relevance of perceived value, trust, and institutional identification to student persistence [43,44]. In Pakistan, institutional reputation and perceived value may also inform students’ educational decisions [35,37]. Nevertheless, the observed association does not establish that loyalty itself produces retention intentions.

The indirect associations provide further insight into the relationships among the study variables. Students’ self-reported knowledge, skills, and competencies and student loyalty separately mediated the association between perceived quality teaching and student retention intention (H7 and H8), while the serial indirect association through perceived knowledge, skills, and competencies and student loyalty was also statistically significant (H9). These findings are consistent with an interpretation in which perceived teaching quality is associated with retention intention through students’ perceived competency development and institutional loyalty. The pattern accords with HCT’s emphasis on capability development and with aspects of EVT that concern students’ evaluations of educational experiences [50,51,83]. However, because expectancy and value were not directly measured, the study does not constitute a direct test of EVT’s central mechanisms. Similarly, the serial indirect association is consistent with a proposed sequence but does not establish temporal ordering or causality. Its interpretation nevertheless complements prior research examining multistage relationships in student-related outcomes [40,48].

Taken together, the results position perceived quality teaching as an important correlate of students’ perceived human-capital development, institutional loyalty, and retention intention. They suggest that teaching quality may be relevant to student persistence through interconnected cognitive and affective associations, rather than through a deterministic process. For HEIs, faculty development, competency-oriented curricula, and supportive institutional experiences warrant attention. These implications should be considered in light of differences in institutional resources, curriculum modernization, and educational conditions across Pakistan. Such contextual factors may influence how students experience teaching and evaluate their institutions; therefore, the findings should not be assumed to apply uniformly across HE settings.

### 6.1 Theoretical contributions

This study contributes to understanding student retention intention by examining the associations among perceived quality teaching, students’ knowledge, skills, and competencies, and student loyalty within a framework informed by Human Capital Theory (HCT) and Expectancy-Value Theory (EVT). Consistent with HCT, the findings highlight the relevance of perceived competency development to student loyalty and retention intention. However, because knowledge, skills, and competencies were self-reported, the results reflect students’ perceptions of their development rather than objectively assessed human capital. The study also identifies student loyalty as an important correlate of retention intention, suggesting that students’ evaluations of their institutional experience may complement their perceptions of learning and competency development. Although EVT provides a useful interpretive perspective, the study does not directly test its central expectancy and value mechanisms because these constructs were not measured. Furthermore, the significant indirect association through students’ self-reported knowledge, skills, competencies and student loyalty provides evidence consistent with a proposed sequence linking perceived quality teaching with retention intention, while the cross-sectional design prevents conclusions about temporal ordering or causality. By examining these relationships in Pakistani HE, the study extends the empirical application of this theoretical framework to a context where institutional resources and educational conditions may shape students’ experiences. Its contribution to the SDG 4 agenda lies in highlighting the relevance of teaching quality, perceived competency development, and institutional loyalty to student retention intention, rather than establishing their effects on actual retention or educational equity.

### 6.2 Empirical evidence

The findings provide empirical evidence of positive associations among perceived quality teaching, students’ knowledge, skills, and competencies, student loyalty, and student retention intention. Perceived quality teaching showed a stronger association with students’ knowledge, skills, competencies than with student loyalty, while student loyalty was positively associated with retention intention. Students’ knowledge, skills, competencies were also positively associated with both student loyalty and student retention intention. The mediation results further identified significant indirect associations through students’ knowledge, skills, competencies and student loyalty separately, as well as through the hypothesized serial mediation pathway. These findings offer empirical support for examining cognitive and affective factors together when investigating student retention intention. However, the results establish neither causal relationships nor a temporal sequence, and the outcome reflects students’ intentions to continue studying rather than actual retention. Moreover, the knowledge, skills, and competencies findings represent students’ self-reported perceptions, not objectively assessed competencies.

### 6.3 Practical/policy contributions

The findings highlight several priorities for HEIs seeking to strengthen student retention intention. HEIs should prioritize faculty development in student-centered teaching practices that support students’ knowledge, skills, and competencies. Investment in appropriate learning infrastructure, digital resources, and labor market–relevant curricula may complement these efforts, although their contribution was not directly examined in this study. Faculty can also support positive institutional experiences through mentoring, constructive feedback, and empathetic communication. Quality assurance units should consider combining student feedback with assessments of demonstrated learning outcomes and measures of student loyalty, rather than relying solely on satisfaction indicators. This approach would help distinguish students’ perceptions of competency development from objectively assessed learning. Policymakers and regulatory agencies, including the Higher Education Commission (HEC), could encourage institutions to monitor perceived teaching quality, learning outcomes, and retention intentions while accounting for differences in institutional resources and student populations. These recommendations align with SDG 4’s emphasis on quality education and equitable learning opportunities. However, the proposed measures should be evaluated over time before their effectiveness in improving actual student retention can be established.

## 7. Conclusion

This study examined the associations between perceived quality teaching and student retention intention, including the proposed mediating roles of students’ knowledge, skills, and competencies and student loyalty, drawing on Human Capital Theory (HCT) and Expectancy–Value Theory (EVT). The findings support the hypothesized pattern of direct and indirect statistical associations, including a significant serial indirect association linking perceived quality teaching with student retention intention through knowledge, skills, competencies and student loyalty. This pattern is consistent with the proposed serial mediation pathway; however, the cross-sectional design does not establish temporal ordering or a sequential causal process. The findings offer context-specific evidence on how students’ perceptions of teaching quality, competency development, and institutional loyalty relate to their intentions to continue studying. Importantly, the outcome examined was retention intention, not actual student retention. Moreover, knowledge, skills, and competencies were self-reported, and the study did not directly measure the central expectancy and value constructs of EVT. The theoretical interpretations should therefore be considered in light of these measurements and design limitations. Future research could use longitudinal designs, objective assessments of learning outcomes, and institutional retention records to examine whether these associations persist over time and correspond to students’ actual continuation of study. Such research could also clarify the applicability of the proposed relationships across different HE contexts.

## 8. Limitations and future research

This study has several limitations that should be considered when interpreting the findings. First, the cross-sectional design does not establish temporal ordering or causality. Although the findings are consistent with the hypothesized serial mediation pathway, reverse or reciprocal relationships among perceived quality teaching, student loyalty, and retention intention cannot be ruled out. Longitudinal research could examine how these relationships develop over time and whether they correspond to actual student retention. Second, although the study relied on self-reported survey data, diagnostic tests, including full collinearity assessment, indicated that common method bias was unlikely to have substantially influenced the findings. However, these diagnostic assessments cannot rule out common-method variance or other sources of measurement error. The relatively high HTMT value between student loyalty and retention intention (.896) also warrants caution regarding their empirical distinctiveness. Nevertheless, future studies could strengthen methodological rigor by incorporating multi-source or objective indicators, such as academic records or institutional tracking systems. Students’ knowledge, skills, and competencies should also be assessed using objective measures of learning outcomes alongside students’ self-reports. Third, the use of convenience sampling at the respondent level may limit the generalizability of the findings, suggesting the need for probability-based sampling and broader institutional coverage in future research. Because students were drawn from 11 universities, their responses may also be clustered within institutions. Future analyses should account for this potential dependence where appropriate. The findings should also be interpreted within the broader institutional context of Pakistani HE. Low public investment and possible curricular misalignment may constrain educational opportunities; however, these factors were not directly examined and should be treated as potential contextual explanations rather than established boundary conditions. Future research could measure institutional conditions and student characteristics to test whether they moderate the proposed relationships. Finally, students’ self-reported knowledge, skills, and competencies were operationalized as a unified latent construct. Although theoretically justified, this aggregation may overlook the distinct contributions of knowledge, skills, and competencies to student retention intention. Future research should examine these dimensions separately and extend the model by incorporating additional psychological and institutional variables, such as academic engagement, perceived institutional support, and employability perceptions. Combining these measures with objective academic outcomes and actual retention records would help establish whether the observed associations extend beyond students’ intentions to continue studying.

## Supporting information

S1 File Survey Data. (XLSX)

S2 File Additional Information. (DOCX)
