# Supplementary material for: Perceived quality teaching and student retention intention: The serial mediation of knowledge, skills, competencies, and student loyalty
Source: PLoS One. 2026 Nov 2;21(11):e0361914. doi: 10.1371/journal.pone.0361914 (PMC13726913; doi:10.1371/journal.pone.0361914)
Supplement: Supplementary file 1 [file pone.0361914.s002.docx]

**Additional Information**

**Ethics Statement**

We, the authors, hereby declare that we have adhered to all ethical practices as delineated and discussed in the ethics approval letter throughout each stage of this study.

**Competing interests**

The authors have declared that no competing interests exist.

**Author Contributions**

The study was conceptualized by SI, with AT, NA and HA contributing to the research design and supporting data screening. SI, AK, and GF conducted the data analysis and drafted the manuscript, while NA, HA and MA supervised the project and reviewed the manuscript. AT, GF, AK and MA verified the analytical methods and results. All authors contributed equally to the discussion of the results and approved the final manuscript.

**Funding**

This research did not receive any specific grant from funding agencies in the public, commercial, or not-for-profit sectors.

**Acknowledgments**

The authors would like to thank the reviewers and the editor for their insightful comments and suggestions.

**Data Availability Statement**

All relevant data are within the manuscript and its Supporting Information files.
